# Association of age-related declined renal function and osteoporosis based on trabecular bone score in Bushehr Elderly Health (BEH) program

**DOI:** 10.1186/s12882-023-03280-5

**Published:** 2023-07-28

**Authors:** Mehdi Mahmudpour, Narges Homayoun, Iraj Nabipour, Mohammad Reza Kalantar Hormozi, Saeid Najafpour boushehri, Bagher Larijani, Afshin Ostovar, Azam Amini, Maryam Marzban

**Affiliations:** 1 The Persian Gulf Tropical Medicine Research Center, The Persian Gulf Biomedical Sciences Research Institute, Bushehr University of Medical Sciences, Bushehr, Iran; 2 Student Research Committee, Bushehr University Of Medical Sciences, Bushehr, Iran; 3 Department of Internal Medicine, School of Medicine, Bushehr University Of Medical Sciences, Bushehr, Iran; 4 The Persian Gulf Marine Biotechnology Research Center, The Persian Gulf Biomedical Sciences Research Institute, Bushehr University of Medical Sciences, Bushehr, Iran; 5 Clinical Research Development Center, The Persian Gulf Martyrs, Bushehr University of Medical Science, Bushehr, 7514763448 Iran; 6 Osteoporosis Research Center. Endocrinology and Metabolism Clinical Sciences Institute. Endocrinology and Metabolism Research Institute, Tehran University of Medical Sciences, Tehran, Iran; 7 Statistical Genetics Lab, QIMR Berghofer Medical Research Institute, Brisbane, QLD Australia

**Keywords:** Osteoporosis, Age-related renal failure, Trabecular bone score, Elderly

## Abstract

**Purpose:**

Osteoporosis is a systemic disease characterized by decreased bone strength and an increased risk of fracture in old age. Age and pathologic renal failure are independent risk factors for osteoporosis. However, it is not determined whether age-related decreased renal function, in the context of senescence, can be considered as an independent risk factor for osteoporosis. Therefore, this study was conducted to evaluate the effect of senescence-induced renal failure on bone quality and trabecular bone score.

**Methods:**

This study used a cross-sectional design and was carried out based on data collected during the Bushehr Elderly Health (BEH) program, Phase II. A total of 2,125 elderly participants aged over 60 years old entered the study after meeting the inclusion criteria and providing informed consent. They underwent examinations for weight, height, abdominal and hip circumference, as well as blood pressure measurement. All participants also underwent DXA to assess bone mass density (BMD). Trabecular bone score (TBS) was calculated using the DXA apparatus software output. Univariate and adjusted multivariate linear regression analyses were used to evaluate the associations.

**Results:**

In the univariate linear regression analysis, there was a direct correlation between age-related renal failure and TBS (β = 0.038, p < 0.0001), neck of femur BMD (β = 0.047, p < 0.0001), and lumbar BMD (β = 0.055, p < 0.0001). However, after adjusting for BMI, age, sex, smoking, and physical activity, no significant association was observed for these variables.

**Conclusion:**

It is hypothesized that age-related renal failure cannot be considered as an independent risk factor for osteoporosis in elderly individuals aged over 60 years old.

## Introduction

In recent years, there has been a significant increase in the geriatric population due to the rise in life expectancy and average lifespan. This population experiences individualized age-related medical problems, such as osteoporosis. Osteoporosis, which is the primary cause of minimal trauma fractures in elderly individuals, is characterized by reduced bone strength and density, defined as 2.5 standard deviations below that of young adults [1]. Numerous etiologies have been identified as proven risk factors for osteoporosis in the elderly, including female gender, smoking, alcohol consumption, and certain medical conditions such as chronic kidney disease, which is a major risk factor for fracture and decreased bone mineral density in the hip [2].

While previous studies have confirmed the association between reduced kidney function and decreased bone mineral density (BMD) and bone loss [3, 4], it remains unclear whether senescence-related decline in renal function in the absence of other well-known causes of renal failure can have similar effects on BMD and bone loss.

The kidneys, like other organs, undergo certain physiological and structural changes during the aging process that may not be distinguishable from other secondary causes of structural and physiological alterations [5]. There is evidence suggesting a possible association between age-related physiological changes in the kidney and decreased BMD. During kidney senescence, decreased activity of renal 1α-hydroxylase, along with diminished calcium absorption ability in the intestine, results in secondary hyperparathyroidism and bone loss [6]. Additionally, senescence leads to decreased expression of the vitamin D receptor and Transient Receptor Potential cation channel subfamily V 5 (TRPV5) in the kidney, resulting in lower calcium reabsorption in renal tubules [7].

Although the current method for assessing fracture risk in patients with CKD remains DXA [1] it appears that TBS may also have a significant role in evaluating patients at risk of osteoporosis [8, 9], The association between TBS and fracture risk is independent of BMD and other clinical risk factors in individuals with both normal and reduced kidney function [10]. However, these findings have been observed in models of secondary and pathological kidney failure, which typically occur due to other diseases.

Although available physiological evidence may suggest a potential association between age-related declined renal function as an independent risk factor for bone loss, there is conflicting report regarding the attenuation of this association in very old age (3). Therefore, this study was conducted to investigate the association between age-related decline in renal function and BMD, as well as bone quality based on the trabecular bone score, taking into account the effects of other factors in the elderly population of the BEH program.

## Materials and methods

### Study population

The protocols for phases one and two of the BEH program in this study were previously published [11]. This study was conducted using a cross-sectional design based on data obtained during the Bushehr Elderly Health Program (BEH), which is an ongoing prospective population-based cohort study. The study sample consisted of elderly individuals aged 60 years or older residing in the urban population of Bushehr city, which is located in the north part of the Persian Gulf. The primary objective of the cohort was to determine the prevalence and risk factors of non-communicable diseases (NCDs).

### Data collection

Trained interviewers collected data on demographic information, physical activity, and smoking consumption. Demographic and lifestyle data were collected using a standard self-reported questionnaire [6, 7]. The Global Physical Activity Questionnaire (OPAG) was used to assess physical activity, and a brief Tobacco Questionnaire was used to collect information on tobacco smoking consumption. Tobacco smoking consumption was categorized into three groups: nonsmokers had no experience of hookah or cigarette consumption, past smokers had a history of tobacco smoking but were not currently using tobacco at the time of the study, and current smokers were actively smoking cigarettes or hookahs either regularly or occasionally.

The physical activity level during work, sports, and leisure time was calculated based on metabolic equivalents. Lifestyle was classified into five categories: no activity, sedentary (1-1.39), low active (1.4–1.59), active (1.6–1.89), and very active (1.9–2.5) [10].

Anthropometric measurements, including height and weight, were performed according to The Third National Health and Nutrition Examination Survey (NHANES III) anthropometric measurement protocol. Body mass index (BMI) was calculated using the formula weight (kg) / [height (m)]^2 [12].

Medical Examination Qualified physicians conducted medical examinations, and the findings were recorded in a file assigned to each participant. Fasting blood samples were collected after an overnight fasting period of 8–12 h. Commercial kits (Pars Azmoon, Karaj, Iran) were used to measure fasting plasma glucose (FPG), calcium (Ca), phosphorus (P), uric acid, and creatinine (Cr) with an auto-analyzer.

Bone mineral density (BMD) measurements of the femoral neck, total hip, and lumbar spine (L1-L4) were obtained using DXA (Hologic Inc., USA). The TBS iNsight® software installed on the DXA machine was used to calculate the L1-L4 TBS.

Definitions The estimated glomerular filtration rate (eGFR) was calculated using the Modification of Diet in Renal Disease (MDRD) formula based on plasma creatinine: 175 × (Cr)^-1.154 × (age)^-0.203 × 0.742 (if female) × 1.210 (if black race) [13].

## Inclusion and Exclusion Criteria

The inclusion criteria for participants were as follows: age over sixty years, residence in Bushehr city for at least one year before the study, no plans to move or leave the city, sufficient ability to participate in the program, and completion of the informed consent form. Male and female subjects were excluded if they had serum Cr levels ≥ 1.3 and 1.1, respectively, a positive history of diabetes mellitus, or uncontrolled hypertension.

Ethical Considerations This study received approval from the Ethical Research Committee (ERC) of the Endocrinology and Metabolism Research Institute and the ERC of Bushehr University of Medical Sciences. Informed consent was obtained from all participants. (Ethical Code: IR.TUMS.EMRI.REC.1394.0036)

Statistical Analysis The normal distribution of continuous variables was assessed using the Kolmogorov-Smirnov test. Descriptive statistics were presented as mean ± SD for continuous variables, and number and percentage for qualitative data. A p-value less than 0.05 was considered statistically significant.

To assess the differences between definitions of osteoporosis (based on BMD and TBS) and eGFR status (between 45 and 60 and above 60), univariate and multivariate linear regression models were used. In the multivariate analysis, three models were employed: the first model included age and sex; the second model included age, sex, physical activity, and current smoking status; and the final model included age, sex, physical activity, current smoking status, and BMI. The selection of variables was based on a p-value less than 0.05 in the univariate model and clinical implications from the literature. All statistical analyses were performed using STATA software (Release 12, StataCorp LP, College Station, Texas).

## Results

The mean age of the participants was 68.83 ± 6.04 years, of which 54.44% were female. Among the total participants, 405 (21.66%) were using current cigarettes or hookah, while 897 (47.97%) reported past consumption of tobacco. Only about 7% of the elderly people were active, while the other participants had a sedentary lifestyle, low activity, or no activity. The general characteristics of the participants according to their eGFR status are illustrated in Table 1.

**Table 1:** General characteristics of the participants based on their eGFR status

		Total	eGFR = 45–60	eGFR > 60
		Mean ± SD	Mean ± SD	Mean ± SD
**BMI**		27.42 ± 4.79	28.24 ± 5.08	27.28 ± 4.73
**Age**		68.83 ± 6.04	71.2 ± 7.13	68.41 ± 5.71
**Calcium**		9.35 ± 0.52	9.46 ± 0.54	9.33 ± 0.51
**Phosphor**		4.01 ± 0.55	4.11 ± 0.50	3.99 ± 0.56
**Uric Acid**		4.91 ± 1.10	5.17 ± 1.02	4.87 ± 1.11
**Sex**		N (%)	N (%)	N (%)
	**Female**	1,018 (54.44%)	227 (79.65%)	789 (50.00%)
	**Male**	852 (45.56%)	58 (20.35%)	789 (50.00%)
**smoking**	**None**	568 (30.37%)	85 (29.82%)	481 (30.48%)
	**Past-cigarette or hookah**	897 (47.97%)	137 (48.07%)	757 (47.97%)
	**current cigarette or hookah**	405 (21.66%)	63 (22.11%)	340 (21.55%)
**Physical activity**	**< 1 = NO activity**	104 (5.56%)	32 (11.23%)	72 (4.56%)
	**1-1.39 = Sedentary**	1,313 (70.21%)	189 (66.32%)	1121 (71.04%)
	**1.4–1.59 = Low active**	325 (17.38%)	43 (15.09%)	279 (17.68%)
	**1.6–1.89 = Active**	107 (5.72%)	20 (7.02%)	86 (5.45%)
	**>=1.9 = Very active**	21 (1.12%)	1 (0.35%)	20 (1.27%)

As demonstrated in Table 2, the neck of femur BMD, lumbar BMD, and the L1-L4 TBS were significantly higher in the eGFR > 60 group compared to the eGFR 45–60 group.

**Table 2:** Bone health status of the individual based on their MDRD status

	eGFR = 45–60	eGFR > 60	P-value
Neck of femur BMD mean (SD) gr/cm^2^	0.614 ± 0.134	0.661 ± 0.141	< 0.0001
Lumbar BMD mean (SD) gr/cm^2^	0.838 ± 0.160	0.893 ± 0.180	< 0.0001
TBS (L1-L4) mean (SD)	1.259 ± 0.102	1.298 ± 0.103	< 0.0001

Univariate linear regression results revealed that a eGFR greater than 60 had a significant positive association with neck of femur BMD (β = 0.047, P < 0.0001). However, this association was not significant in the multivariable regression model after adjusting for age and sex (β = -0.015, P = 0.052). Moreover, univariate linear regression results showed a significant positive association between eGFR > 60 and lumbar BMD (β = 0.055, P < 0.0001), but this association was not significant after adjusting for age and sex (β = -0.009, P = 0.358). The TBS L1-L4 also showed a significant association in the univariate model (β = 0.038, P < 0.0001), but it was not significant after adjusting for age and sex (β = -0.002, P = 0.718), and in the fully adjusted model (after adjustment for age, sex, physical activity, and current smoker, BMI), the beta decreased to the lowest level (β = -0.003, 95% CI: -0.015 to 0.007). Refer to Table 3 for more details.

**Table 3:** Linear association between TBS, BMD with MDRD status

	Neck of femur BMD mean		Lumbar BMD mean		TBS (L1-L4) mean ± SD	
Univariate Model	Beta (95% CI)	P-Value	Beta (95% CI)	P-Value	Beta (95% CI)	P-Value
**eGFR = 45–60**	Reference	Reference	Reference	Reference	Reference	Reference
**eGFR > 60**	0.047 (0.029–0.064)	< 0.0001	0.055 (0.032–0.077)	< 0.0001	0.038 (0.025–0.052)	< 0.0001
*First Multivariate Model						
**eGFR = 45–60**	Reference	Reference	Reference	Reference	Reference	Reference
**eGFR > 60**	-0.015(-0.030–0.0001)	0.052	-0.009 (-0.029–0.010)	0.358	-0.002 (-0.013–0.009)	0.718
**second Multivariate Model						
**eGFR = 45–60**	Reference	Reference	Reference	Reference	Reference	Reference
**eGFR > 60**	-0.014 (-0.029–0.001)	0.072	-0.007 (-0.028–0.012)	0.464	-0.001 (-0.013- 0.009)	0.750
**Final Multivariate Model						
**eGFR = 45–60**	Reference	Reference	Reference	Reference	Reference	Reference
**eGFR > 60**	-0.010 (-0.025–0.004)	0.172	-0.001 (-0.020–0.017)	0.860	-0.003 (-0.015–0.007)	0.533

*Adjusted for age and sex

**Adjusted for age, sex, physical activity, and current smoker

***Adjusted for age, sex, physical activity, and current smoker, BMI

## Discussion

Based on our knowledge, this is the first community-based cross-sectional study that evaluates the association between TBS and renal function status in the elderly Iranian population. The mean values of femoral neck and lumbar BMD and TBS L1-L4 were significantly higher in participants with eGFR > 60 compared to those with eGFR 45–60. Univariate linear regression results revealed a significant positive association between neck of femur BMD, lumbar BMD, and L1-L4 TBS with higher eGFR. However, in multivariate analysis, the associations between these variables were not significant after fully adjusting for age, sex, physical activity, smoking status, and BMI.

Cumulative evidence has confirmed the association between chronic kidney disease and osteoporosis, as well as an increased risk of fracture [14, 15]. This association may be related to pathophysiological alterations in chronic kidney disease, such as secondary hyperparathyroidism, altered metabolism of the active form of vitamin D, phosphorus retention, chronic metabolic acidosis, increased levels of sclerostin and FGF23. These factors play a pivotal role in bone mineral metabolism [16]. Therefore, it can be hypothesized that age-related decline in renal function may result in decreased BMD in healthy elderly individuals. However, according to the results of the current study, this hypothesis failed to be confirmed.

Li et al. revealed a positive association between decreased renal function and lower BMD in postmenopausal healthy women with eGFR > 60. However, the adjusted model based on age, duration of menopause, and BMI failed to reveal a significant association [12]. Another cohort study indicated the association between eGFR and BMD in an average age of 75-year-olds, while in an average age of 80 and 85, the association was not significant (3).

The majority of studies that have indicated the association of renal failure with decreased BMD have been conducted on patients suffering from chronic kidney disease [17]. Moreover, the pathophysiological alterations in renal failure leading to disturbances in bone mineral metabolism almost invariably occur in eGFR < 60 and are not easily distinguishable from primary osteoporosis. The previous results regarding the effects of pathological CKD on BMD may not be easily attributable to bone metabolism in age-related renal failure in healthy elderly individuals. Therefore, in extreme old age, based on contrary results, other factors may modulate or even counteract the expected physiological alteration of bone.

There are some well-known pathological mechanisms involved in the pathogenesis of osteoporosis. It has been elucidated that parathyroid hormone (PTH) increases more in higher stages of renal failure [17]. Carrevick et al. revealed that in extreme old age, PTH levels were still high regardless of eGFR, even in eGFR > 60 [18]. In pathological CKD models, it has been reported that PTH does not have a significant effect on bone metabolism in the extreme eGFR quartiles and there is an attenuated relationship between declined eGFR and fracture risk [19]. Therefore, in healthy elderly individuals, the altered effect of PTH on bone may attenuate the strength of the relationship between eGFR and BMD.

FGF23, as an osteocyte-originated hormone and key regulator of phosphorus and vitamin D, plays an important role in the pathogenesis of osteoporosis and TBS decline [20]. This hormone starts to be secreted when eGFR is less than 60 [21] and is inhibited by klotho. Moreover, klotho is increased in the early stage of renal failure as a compensatory hormone [22]. Therefore, in the current study, in the early stage of renal failure, the possible compensatory increase in klotho may decrease the plasma level of FGF23, attenuate its destructive effect on TBS, and modulate the effect of renal failure on TBS.

The receptor activator of nuclear factor-κB (RANK) pathway is a well-known pathway in the metabolism of bone minerals. The interaction between RANK and its ligand RANKL results in the stimulation of osteoclasts and the production of cytokines [23]. Osteoprotegerin (OPG), as an osteoblast-derived anti-inflammatory protein, inhibits osteoclasts through competitive inhibition of RANK, resulting in decreased bone resorption [24]. The RANKL-OPG imbalance plays a major role in the pathogenesis of osteoporosis and the inflammatory process [25]. Therefore, OPG has been introduced as an anti-osteoporotic agent [26]. It has been demonstrated that OPG increases with aging [27], and there is a eGFR-independent positive correlation between OPG and aging. Vik et al. revealed a significant negative correlation between eGFR and OPG in an average age older than 62.2 [28]. Therefore, it might be hypothesized that increased OPG may have a protective, anti-osteoporotic role in age-related declined renal function.

The major limitation of this study was the lack of measurement of parameters involved in the pathogenesis of osteoporosis, such as PTH, FGF23, OPG, RANK, and RANKL. The contradictory results of the current study necessitate further studies to clarify the exact mechanism of bone metabolism in extreme old age. It can be hypothesized that the well-known pathophysiological mechanisms responsible for pathologic CKD-induced osteoporosis may not act in similar patterns in senescence-induced renal failure in healthy elderly individuals. The physiological alterations in extreme old age may have unknown protective or stimulatory mechanisms that act through other unknown mechanisms in bone mineral metabolism.

## Conclusion

Osteoporosis is a significant problem for the global community. With the aging process, osteoporosis and related fractures have a great impact on the community, and the burden of disease will likely continue to increase. Several early interventions are predicted to decline the mortality, morbidity, and economic impact of age-related osteoporosis. Although previous studies indicate the effect of renal function as an independent risk factor for bone loss, the current study, when considering confounding variables, cannot indicate the direct and independent effect of age-related renal dysfunction on osteoporosis. Future research is needed to determine the pathological pathway of this hypothesis.

## Data Availability

The datasets used during the current study are available from the corresponding author, AO (a.ostovar@bpums.ac.ir) or IN (inabipour@gmail.com), upon reasonable request.
